# An Emerging Role for Post-translational Modifications in Regulating RNP Condensates in the Germ Line

**DOI:** 10.3389/fmolb.2021.658020

**Published:** 2021-04-08

**Authors:** Jennifer A. Schisa, Mohamed T. Elaswad

**Affiliations:** Department of Biology, Central Michigan University, Mount Pleasant, MI, United States

**Keywords:** RNP granules, phase transition, germ line, methylation, phosphorylation, condensate

## Abstract

RNA-binding proteins undergo regulated phase transitions in an array of cell types. The phase separation of RNA-binding proteins, and subsequent formation of RNP condensates or granules, occurs during physiological conditions and can also be induced by stress. Some RNP granules have roles in post-transcriptionally regulating mRNAs, and mutations that prevent the condensation of RNA-binding proteins can reduce an organism’s fitness. The reversible and multivalent interactions among RNP granule components can result in RNP complexes that transition among diffuse and condensed states, the latter of which can be pathological; for example, in neurons solid RNP aggregates contribute to disease states such as amyotrophic lateral sclerosis (ALS), and the dysregulation of RNP granules in human germ cells may be involved in Fragile X-associated primary ovarian insufficiency. Thus, regulating the assembly of mRNAs and RNA-binding proteins into discrete granules appears to provide important functions at both cellular and physiological levels. Here we review our current understanding of the role of post-translational modifications (PTMs) in regulating the condensation of RNA-binding proteins in the germ line. We compare and contrast the *in vitro* evidence that methylation inhibits phase separation of RNA binding proteins, with the extent to which these results apply to the *in vivo* germ line environment of several model systems. We also focus on the role of phosphorylation in modulating the dynamics of RNP granules in the germ line. Finally, we consider the gaps that exist in our understanding of the role of PTMs in regulating germ line RNP granules.

## Introduction

Phase separation is an important principle of cellular organization. Many types of membraneless organelles (MLOs) assemble through the process of liquid-liquid phase separation. Some of the best studied MLOs in the cytoplasm are ribonucleoprotein (RNP) granules composed of RNA and RNA binding proteins, such as stress granules and processing bodies. Much of our understanding of phase separation to date has come from *in vitro* studies. From such studies, we now understand that phase separation is driven mainly by weak interactions between multivalent protein interaction domains or intrinsically disordered low complexity domains (LCDs) (Kato et al., 2012; Li et al., 2012). Since multivalent interaction motifs and the short linear motifs in intrinsically disordered regions and LCDs are often post-translationally modified (Xie et al., 2007; Bah and Forman-Kay, 2016; Chong and Forman-Kay, 2016), in hindsight it is not surprising that post-translational modifications (PTMs) have been revealed as important regulators of phase separation (Itakura et al., 2018; Rhoads et al., 2018). While a diverse array of PTMs can modulate condensates, in this review we focus on the best-studied paradigms: methylation and phosphorylation.

PTMs can alter the chemical properties of amino acids, such as the steric properties, bulkiness, or charge state. For example, when Arginine (Arg) is methylated, bulkiness is increased, and the distribution of charge and hydrophobicity is altered which affects intermolecular interactions and phase separation. Phosphorylation of Tyr or Ser introduces a negative charge which can either promote or inhibit phase separation (Monahan et al., 2017; Wang et al., 2018); thus, PTMs can weaken or enhance multivalent interactions between phase-separated macromolecules. PTMs can also recruit protein into, or exclude protein from, the condensate (Hofweber and Dormann, 2019). Thus, PTMs can modulate the assembly and disassembly of liquid-like RNP granules, and transitions from the liquid state to gel- or solid-like states.

Condensation of RNA binding proteins and RNA in the germ line of many organisms results in germ granules (Voronina et al., 2011). A variety of terms are used to describe the array of germ granules found across different species which can be confusing but are described in several resources (Table 1 and Schisa, 2012). While some germ granule proteins exhibit liquid-like properties, such as the PGL-1 granules in *C. elegans* embryos (Brangwynne et al., 2009), other types of germ granules such as the Balbiani body in *Xenopus* oocytes have solid-like properties (Boke et al., 2016; Woodruff et al., 2018). Careful examination has revealed multiple phases within germ granules; for example, the germ granules of early *C. elegans* embryos include a liquid-like phase of PGL proteins, and a gel phase of MEG-3 protein that appears to have a scaffolding role in the assembly of germ granules (Putnam et al., 2019). An increasing number of examples of PTMs modulating the assembly of germ granules have been documented over the past two decades. Building upon our understanding from *in vitro* studies, these *in vivo* experiments are revealing both conserved and complex roles of PTMs in regulating condensates of RNA binding proteins in the germ line that are associated with critical germ line functions. This review will focus on our understanding of how methylation and phosphorylation regulate germ line RNP condensates across invertebrate and vertebrate model systems.

**TABLE 1:** Summary of germ line RNP condensates described in this review.

**Species**	**Germ granule/RNP condensate**	**Description**
All	Germ granule	Refers collectively to the electron-dense, RNP granules in vertebrate and invertebrate germ lines, often given a specific name in a species.
*Drosophila*		
	Nuage	Perinuclear, small Vasa-positive granules in nurse cells of ovary, and in several stages of spermatogenesis.
	Pole plasm	Posterior cytoplasm of the oocyte that contains polar granules; it is necessary and sufficient for the induction of germ cells.
	piNG body (piRNA nuage giant body)	Granules, larger than nuage, that appear late during spermatogenesis; granules contain several components of the piRNA pathway.
*C. elegans*		
	P granule	Germ granules in adult germ cells and germ cell precursors (P lineage) of embryo; perinuclear during most of development.
Mouse		
	Chromatoid body	Nuage component of mammalian spermatogenic cells; condensed form detected after completion of meiosis; contains similar proteins as germ granules in female germ cells, e.g., mouse Vasa homolog.
	Cajal body	Non-membraneous nuclear organelle; site of spliceosome maturation.
	Stress granule	RNP granules induced by heat stress; detected in spermatogonia and preleptotene and early pachytene spermatocytes.
	Oocyte aggregate	A subcortical RNP aggregate in germinal vesicle-stage oocytes, contains maternal mRNAs, and P body proteins.
Zebrafish		
	Balbiani body	Structure in zebrafish (and other) oocytes analogous to the mitochondrial cloud.
*Xenopus*		
	Oocyte aggregate	Patches of XStau1 in the vegetal subcortical region of Stage VI oocytes and eggs.

## Role of Methylation

### Methylation Inhibits Condensate Assembly in *in vitro* Studies

**FIGURE 1:**
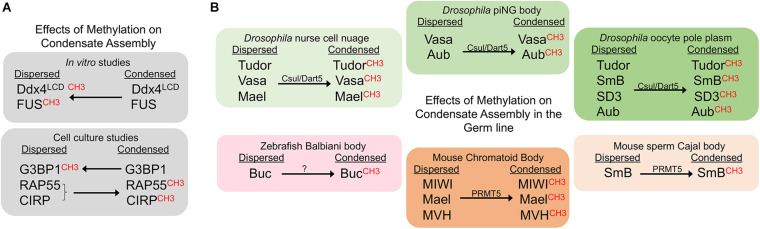
Methylation promotes condensate assembly in the germ line. CH3 indicates the methylated form of the corresponding protein. Arrows indicate whether methylation promotes or inhibits a condensed state. **(A)** Methylation suppresses condensates in *in vitro* studies, but can sometimes promote condensation in cell culture. **(B)** Methylation promotes condensation of germ line proteins. Effects of methylation are shown for six distinct germ line RNP granules in three model systems.

Methylation is a key regulator of phase transitions and RNP granule dynamics. Within many RNP granules are proteins with RGG or RG-rich motifs, and the Arginine residues in these motifs are often methylated by protein arginine methyltransferase (PRMT) enzymes (Bedford and Clarke, 2009). In general, Arg-methylation is considered a less dynamic modification, in how it impacts target proteins, than others such as phosphorylation and acetylation (Fackelmayer, 2005). In *in vitro* studies methylation of Arg weakens intermolecular interactions and thus inhibits phase separation of RNA binding proteins. For example, droplets of the N-terminal RGG-rich domain of the conserved nuage protein Ddx4/Vasa are destabilized by Arg-methylation (Nott et al., 2015). Methylation of recombinant or purified FUS protein, the protein that phase separates into granules in amyotrophic lateral sclerosis (ALS) mutations, similarly reduces liquid-liquid de-mixing (Qamar et al., 2018; Hofweber and Dormann, 2019). Since no examples have shown Arg-methylation to promote condensation *in vitro*, it has been suggested that this PTM is a general inhibitor of Arg-aromatic (π) interactions that reduces phase separations (Hofweber and Dormann, 2019). However, *ex vivo* studies reveal a more complex effect of methylation on phase separation (Figure 1A). Some experiments align with *in vitro* results showing Arg-methylation suppresses RNP granule formation. Treatments of cultured U2OS cells that increase the methylation of Ras-GAP SH3-binding protein (G3BP1) repress the assembly of stress granules (Tsai et al., 2016). However, there is also evidence for Arg-methylation promoting RNP granule assembly. When methylation of the Lsm4 protein, RAP55A, is decreased, the localization of RAP55A to P bodies is inhibited in cell culture (Matsumoto et al., 2012), and similarly, recruitment of unmethylated CIRP to stress granules is blocked (De Leeuw et al., 2007). Overall, many *in vitro* and *ex vivo* examples highlight a role for Arg-methylation in controlling the dynamics of RNP granules; however, these results do not address the extent to which this regulation occurs *in vivo* or in the germ line. To date, studies in three model systems all demonstrate a role for Arg-methylation in promoting phase separation of RNA binding proteins in the germ line, opposite of the role seen *in vitro* (Figure 1B).

### Methylation Promotes Condensate Assembly in the Germ Line

In *Drosophila*, components of the methylosome regulate RNA binding proteins in multiple MLOs of the female germ line (Figure 1B). Capsuleen (Csul), also known as Dart5, is the homolog of the methyltransferase PRMT5. In *dart5/csul* mutant egg chambers the condensation of Tudor, Vasa, and Maelstrom into perinuclear granules of the nurse cell nuage is diminished, suggesting methylation normally promotes condensation of these proteins in the *Drosophila* female germ line (Gonsalvez et al., 2006; Anne et al., 2007). The Capsuleen-Valois methylosome complex also has a role in assembly of the pole plasm of *Drosophila* oocytes. The localization of Tudor and the Sm proteins, SmB and SD3, to the posterior pole plasm requires the methylation of Arg residues (Anne et al., 2007; Anne, 2010). The consequence of blocked methylation and disrupted nuage and pole plasm assembly in *dart5/csul* mutants is a grandchildless phenotype, where embryos of mutant females completely lack pole cells and develop into agametic, sterile adults (Gonsalvez et al., 2006; Anne et al., 2007). Csul also methylates the Piwi protein Aubergine (Aub), which is required for Aub to bind Tudor and to promote the assembly of pole plasm in the developing oocyte (Kirino et al., 2010).

A role for methylation has also been identified in *Drosophila* primary spermatocytes, where a novel condensate called the piRNA nuage giant body (piNG-body) is enriched for Vasa, Aub, Argonaute 3, and Tudor (Kibanov et al., 2011). In spermatocytes lacking Csul/PRMT5, unmethylated Vasa and Aub fail to condense into the piNG-body; only small Vasa-positive nuage granules, and unlocalized Aub signals are detected. At the same time, the piRNA pathway is disrupted, and male sterility occurs. Thus, methylation by PRMT5 appears to be essential for piNG-body assembly and normal development of the male germ line.

Methylation of Ddx4/Vasa is widely conserved from planar worms to humans (Rouhana et al., 2012); thus, it will be interesting to determine if Arg-methylation also promotes assembly of Vasa granules in systems beyond *Drosophila*. It is notable that these *in vivo* germ line studies show an opposite effect of methylation as compared to *in vitro* studies, where PRMT1-dependent methylation disrupts the phase separation of Ddx4 (Nott et al., 2015). This difference seems likely to be due to the fact *in vitro* studies generally involve only one or a few purified RBPs while the *in vivo* environment is much more complex.

Piwi-tudor domain protein interactions promote the assembly of germ granules not only in *Drosophila*, but also in mouse (Arkov and Ramos, 2010). *In vitro* studies show that Tudor proteins recognize methylarginine marks on mouse Piwi proteins to drive their localization to cytoplasmic foci (Vagin et al., 2009). In cell culture, treatment with an inhibitor of methyltransferases abolishes interactions between Tdrd1 and the mouse Piwi protein, MILI, suggesting that Arg-methylation and Tudor binding promote assembly of piRNA pathway components into nuage (Vagin et al., 2009). Moreover, immunoprecipitation studies show Tdrd6 interacts with the mouse Piwi proteins Miwi and Mili *in vivo*, and Miwi is methylated by PRMT5 and binds Tdrd6 in a symmetrical dimethylarginine methylation (sDMA)-dependent manner (Vasileva et al., 2009; Kirino et al., 2010). In *tdrd6-/-* testes the RNA binding proteins Mael, Miwi, and Mouse Vasa homolog (MVH)/Ddx4 fail to condense into the normal chromatoid bodies (the nuage in mouse spermatogenic cells) (Vasileva et al., 2009). The defects in condensation are accompanied by a lack of elongated spermatids and sperm. Given the proposed role for chromatoid bodies as storage sites during spermatid differentiation, their aberrant architecture and absence of condensed Mael, Miwi, and MVH in chromatoid bodies may directly impact spermatid differentiation (Vasileva et al., 2009). PRMT5 also methylates the SmB splicing protein in mouse spermatocytes. When Arg-methylation is abrogated via mutation of Tdrd6, the assembly of spliceosomes is impaired in primary spermatocytes, resulting in a decreased number of Cajal bodies, the nuclear membraneless condensates where spliceosome maturation occurs (Akpınar et al., 2017). Thus, methylation promotes condensation in both cytoplasmic and nuclear compartments of the mouse male germ line.

In the zebrafish model system methylation appears to promote the condensation of a solid-like germ granule, the Balbiani body. The germ plasm in early embryos originates from the Balbiani body in the oocyte (Kloc et al., 2004). A role for Tudor6 (Tdrd6) has been shown in modulating the aggregation of Buckyball (Buc), the organizer of the Balbiani body (Roovers et al., 2018). Tdrd6a and Tdrd6c interact with Buc via its three symmetrically dimethylated arginines. The three arginines of Buc are required for Buc to condense into a mature Balbiani body in oocytes, and to form germ plasm in embryos. The importance of Arg-methylation in promoting this phase transition to a solid condensate is further underscored by the observation that deleting the three arginines of Buc has a more severe phenotype than a *tdrd6a* mutant, with defects in germ cell formation and embryonic development (Roovers et al., 2018). It will be interesting to determine if methylation also modulates condensation of germ granule proteins in the adult germ cells or embryonic primordial germ cells where Tdrd7 has a role in maintaining the integrity of the germ granule protein Vasa (Strasser et al., 2008). The recent discovery of a role for PRMT5 in the methylation of Zili and Vasa in the zebrafish gonad should allow researchers to address whether methylation has any role in modulating condensation of Zili, Vasa, or other granule components (Zhu et al., 2019).

No studies to date have identified regulation of RNP condensates by methylation in the *C. elegans* germ line. However, Arg-methylation of the *C. elegans* RG proteins PGL-1/-3 by the PRMT1 homolog EPG-11 results in decreased phase separation *in vitro* (Zhang et al., 2018). In addition, in *C. elegans* embryos, where the primordial germ cells localize PGL-1/-3 to germ granules, EPG-11 destabilizes PGL-1/-3 aggregates from somatic blastomeres via methylation of the PGL-1/-3 RGG repeats (Li et al., 2013). It remains to be determined if this example of an inhibition of condensation by methylation will be extended to the worm germ line in future studies. In any event, the experiments in the fly, fish, and mouse germ lines clearly indicate differences from how methylation modulates phase separation *in vitro* and highlight the necessity of additional *in vivo* studies. Biochemical approaches to further study Ddx4/Vasa may be especially valuable due its broad conservation. Employing a high-resolution mass spectrometry approach to profile PRMT substrates may also be useful, as has been successful in other contexts (Shishkova et al., 2017).

## Role of Phosphorylation

### Phosphorylation Can Promote or Inhibit Condensate Assembly *in vitro*

**FIGURE 2:**
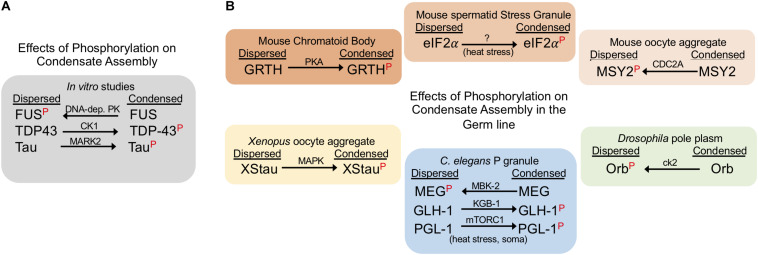
Phosphorylation can suppress or promote condensate assembly. The red P indicates the phosphorylated form of the corresponding protein. Arrows indicate whether phosphorylation drives a dispersed or condensed state; kinase names are above each arrow. **(A)** Phosphorylation has varying effects on phase separation in *in vitro* studies. **(B)** Phosphorylation negatively and positively regulates RNP condensates in the germ line. The effects of phosphorylation are shown for proteins in six distinct germ line RNP granules in three model systems. Note: phosphorylation of the *C. elegans* P granule protein PGL-1 during heat stress conditions promotes condensates in somatic cells, outside of the germline.

Phosphorylation is a common PTM that is implicated in the regulation of RNP granule dynamics. Phosphorylation is a rapid and reversible process by which proteins acquire negatively charged PO_4_ groups that alter their intramolecular interactions and consequently impact phase separation (Hofweber and Dormann, 2019). Multivalent interactions among serine and tyrosine residues are especially prominent in the LCDs and intrinsically disordered regions (IDRs) of RNA binding proteins in granules. In *in vitro* studies phosphorylation sometimes promotes, and other times suppresses, phase separation of RNA binding proteins (Hofweber and Dormann, 2019). For example, phase separation of FUS is blocked when FUS is phosphorylated by the DNA-dependent protein kinase (Monahan et al., 2017; Murray et al., 2017; Luo et al., 2018). In contrast, phosphomimetic S48E substitution in the N-terminal domain of TDP-43 (TAR DNA– binding protein of 43 kDa) blocks the phosphorylation of TDP-43 by Casein Kinase 1 (CK1), and leads to reduced liquid-liquid phase separation of TDP-43 *in vitro* (Kametani et al., 2009; Wang et al., 2018; Hofweber and Dormann, 2019). Phosphorylation also promotes the condensation of Tau, a neuron-specific microtubule-associated protein (Ambadipudi et al., 2017). Similar to the varied effects of phosphorylation in *in vitro* studies (Figure 2A), cell culture experiments also reveal both inhibitory and stimulatory roles of phosphorylation on condensation of stress granule proteins (Hofweber and Dormann, 2019). It is not yet clear how well the *in vitro* and *ex vivo* studies translate to more complex environments of *in vivo* tissues and organisms. However, the studies highlighted below elucidate a growing role for phosphorylation in regulating the assembly and disassembly of germ granules in invertebrate and vertebrate systems.

### Phosphorylation Can Promote or Inhibit Condensate Assembly in the Germ Line

Studies of *C. elegans* germ cells reveal insights into the complex roles of phosphorylation in regulating RNP granule assembly (Figure 2B). MBK-2, the *C. elegans* DYRK3 kinase homolog, and the PP2A^*PPTR–*1/2^ phosphatase are key players in mediating the dynamics of PGL-1, an RGG protein in germ granules (Seydoux, 2018). Phosphorylation of MEG-1 and MEG-3 (maternal-effect germline defective) proteins by MBK-2 promotes disassembly of PGL-1 granules in zygotes (Wippich et al., 2013). This action is balanced by PPTR-1 and PPTR-2 which have redundant phosphatase functions to dephosphorylate MEG proteins and stabilize PGL-1 granule formation (Wang et al., 2014). In addition to contributing to P granule regulation, the MEG proteins are required for fertility; however, how regulated phosphorylation impacts fertility is not yet clear (Wang et al., 2014). A contrasting example of phosphorylation in *C. elegans* is the Ddx4/Vasa homolog GLH-1 which is phosphorylated by the KGB-1 MAP kinase (Orsborn et al., 2007). In the *kgb-1* knockout the localization of GLH-1 to discrete P granules is partially disrupted, suggesting that KGB-1 normally promotes condensation of GLH-1 via phosphorylation. When GLH-1 is not phosphorylated by KGB-1, elevated levels of GLH-1 protein are detected in the gonad, as well as over-proliferation of germ cells, and a high level of sterility. A second example of phosphorylation promoting condensation of P granules has been described outside of germ cells. mTORC1-mediated phosphorylation of PGL-1/-3 during mild heat-stress promotes the assembly of PGL-1/-3 granules in somatic blastomeres of early embryos (Zhang et al., 2018).

In the *Drosophila* germ line, phosphorylation has been shown to inhibit protein condensation. Orb, the cytoplasmic polyadenylation element binding protein (CPEB) homolog, regulates the translation of target mRNAs in *Drosophila* ovaries and is phosphorylated by casein kinase II (ck2) (Wong et al., 2011). When ck2 activity is compromised, Orb transitions from a diffuse state in oocytes into condensed sponge body-like granules, implicating phosphorylation in inhibiting Orb condensation (Wong et al., 2011). Deducing the precise consequence of ectopic Orb condensates in *ck2* mutants is not straightforward; however, the phenotype of both *orb* and *ck2* mutants includes dorsal-ventral defects during oogenesis. Interestingly, several examples demonstrate that phosphorylation does not always affect protein condensation. The *Drosophila* Pan Gu (PNG) kinase directly phosphorylates two de-capping activator proteins Trailer hitch (TRAL)/RAP55 and Me31B/RCK; however, the dispersal of TRAL granules is not affected during *in vitro* egg activation (Hara et al., 2018). In addition, the phosphorylation of maelstrom (Mael) by polo kinase is not required for its localization to nuage puncta in ovaries (Pek et al., 2012).

Multiple examples in both female and male germ lines of vertebrates demonstrate a role for phosphorylation in regulating protein condensation. In the female mouse germ line, MSY2 is one of several RNA-binding proteins that condense into transient, RNA-containing aggregates in fully grown oocytes but later decondense during oocyte maturation (Flemr et al., 2010). Phosphorylation of MSY2 by CDC2A occurs during oocyte maturation concomitant with its de-condensation, suggesting that phosphorylation may inhibit MSY2 condensation (Medvedev et al., 2008). In contrast, in the male mouse germ line, when Gonadotropin-regulated testicular RNA helicase (GRTH) is not phosphorylated by Protein Kinase A (PKA), GRTH localization to the chromatoid body (CB) is impaired, the CB is reduced in size, testis size is reduced due to germ cell apoptosis, and spermatogenesis arrests at the round spermatid stage (Sheng et al., 2006; Kavarthapu et al., 2019). Phosphorylation of eIF2α by GCN2 also appears to promote its condensation into stress granules in male spermatids in response to temperature stress (Kim et al., 2012; Yoon et al., 2017). In *Xenopus* oocytes, Staufen proteins (XStau1 and XStau2) are phosphorylated via the MAP Kinase pathway during meiotic maturation (Allison et al., 2004). Phosphorylated XStau1 appears to transition from small aggregates concentrated locally in the vicinity of the ER, to larger aggregates less localized to the ER, suggesting phosphorylation promotes condensation of XStau1 which may impact its association with the ER network (Allison et al., 2004). It is not yet clear how phosphorylation modifies XStau1 function.

Overall, the role of phosphorylation in modulating phase transitions in the germ line has been understudied to date. Dozens of RNA binding proteins have been characterized as components of P granules in *C. elegans*, and of polar granules and nuage in *Drosophila* (Updike and Strome, 2010; Kato and Nakamura, 2012). It will be interesting to determine the extent to which phosphorylation regulates condensation of these proteins. For example, the *C. elegans* MPK-1 ERK (extracellular signal-regulated kinase) controls seven different processes in the adult germ line, including germ cell apoptosis and oocyte maturation (Lee et al., 2007). Genomic approaches have identified 30 ERK substrates including several RNA binding proteins, some of which are P granule proteins (Arur et al., 2009). Future studies should be able to address if ERK modulates condensation of any of its protein substrates.

## Perspectives

The germ line has distinct functions from the soma, including the need to accurately transmit genetic information between generations and the requirement of pluripotency. The germ line may also have unique requirements in regulating gene expression, as large pools of maternal mRNAs accumulate in oocytes, many of which are non-translating until after fertilization. Germ line RNP condensates can facilitate post-transcriptional gene regulation of mRNA, and the prevailing theory is that such gene regulation is advantageous for rapid gene activation post-fertilization. PTMs afford some of the fastest changes to protein function that are also reversible and avoid *de novo* nuclear activities. Since RNP condensates are very dynamic complexes, these advantages may begin to answer why PTMs have evolved as an important regulator of germ line RNP condensates.

In comparison to the many examples of PTMs modulating phase transitions *in vitro* and in cell culture, there are notably fewer documented cases in the germ line. This discrepancy may simply reflect the challenge of studying PTMs *in vivo*, with the expectation that additional examples will be identified in the future. Alternatively, the relatively low number of examples may be due to alternative mechanisms regulating condensates in the germ line, with a smaller relative role for PTMs. This review highlights one major difference between *in vitro* studies that demonstrate a role for methylation in reducing phase separation, and germ line studies that show a role in promoting condensation of RNA binding proteins. In particular, the example of Ddx4/Vasa is striking. The formation of liquid droplets of the disordered N-terminal domain of Ddx4 is suppressed by asymmetric demethylation via PRMT1 expression in bacterial cells (Nott et al., 2015). In contrast, the methylation of Vasa by Dart5/Csul in *Drosophila* promotes the assembly of nuage granules in nurse cells and of piNG bodies in spermatocytes (Kibanov et al., 2011). The explanation for these opposite effects may simply be a more complex *in vivo* environment in the germ line, but probing this difference could be helpful in understanding the limitations of applying *in vitro* findings to the germ line. Another instance of context-specific effects of PTMs can be seen in the *C. elegans* embryo. MBK-2 phosphorylation of the MEG proteins drives disassembly of P granules, including the PGL-1 protein (Wang et al., 2014); however, phosphorylation of PGL-1 by mTORC1 stimulates the assembly of ectopic P granules in somatic blastomeres during heat stress (Zhang et al., 2018). These differences highlight our incomplete understanding of the role of PTMs in regulating phase separation.

One important consideration not yet discussed is the combinatorial nature of PTMs in modulating the assembly of granules. RNA binding proteins are often both phosphorylated and methylated, suggesting these two types of PTMs can be either synergistic or antagonistic (Bah and Forman-Kay, 2016). In addition, other PTMs such as O-linked GlcNAc modification and lysine acetylation have been shown to interact with phosphorylation in *in vitro* assays. For example, acetylation of Tau on Lys-321 prevents phosphorylation of a downstream Ser residue, and results in decreased aggregation of Tau filaments (Carlomagno et al., 2017). In regards to the germ line, few combinatorial PTMs of RNA binding proteins have been identified to date. However, the *C. elegans* PGL-1 protein is methylated by EPG-11 which inhibits PGL-1 aggregation into granules *in vitro*, and may act similarly in somatic blastomeres of the early embryo (Li et al., 2013; Zhang et al., 2018). The de-condensation of PGL-1 in somatic blastomeres can be balanced during heat stress when phosphorylation of PGL-1 by mTORC1 accelerates phase separations, resulting in ectopic somatic granules (Zhang et al., 2018). It remains to be seen if RNP condensates in the germ line proper are similarly modulated by combinations of PTMs.

Another interesting avenue to pursue is the extent to which stress conditions trigger PTMs that regulate RNP condensates in the germ line. Heat stress, extended meiotic arrest, starvation, hypoxia, and osmotic stress can induce the assembly of large RNP condensates in the germ line; however, PTMs have not been identified as regulators of any of these stress-induced granules to date (Schisa, 2014). *In vitro* studies, on the other hand, show that certain stresses lead to phosphorylation of FUS in addition to its constitutive Arg-methylation (Rhoads et al., 2018). During environmental stresses, stress granule formation is stimulated by a combination of phosphorylation and *O*-GlcNAcylation on Ser/Thr residues; however, the precise cause and effect relationship between these combinations of PTMs and phase transitions remains incompletely understood (Hofweber and Dormann, 2019).

Another unresolved question is whether RNP condensates in germ cells are bona fide phase-separated structures. To address this, time-lapse microscopy could be useful to determine the behavior of fusing droplets *in vivo*. FRAP studies may also be helpful; however, fast FRAP recovery is not sufficient to demonstrate phase separation (Alberti et al., 2019). The use of super-resolution microscopy is a relatively new tool that may be useful in mapping phase diagrams to determine concentration dependent thresholds for assembly (Patel et al., 2015; Rai et al., 2018). Another approach being developed to assess viscosity and porosity of condensates is the use of genetically encoded nanoparticles (GEMS) as microrheology probes (Alberti et al., 2019). One challenge to the field with investigations of the physical properties of *in vivo* germ line condensates is distinguishing whether genetic perturbations that affect function do so due to altered condensation or independently of condensation alterations.

We have a good appreciation that the dysregulation of protein folding and condensation can result in human disease, as is well-exemplified by multiple neurodegenerative diseases, for example, ectopic aggregates containing TDP-43 and FUS in ALS. Germ cells share several attributes with neurons such as being post-mitotic and differentiated, and relying on regulation of gene expression post-transcriptionally, e.g., the synaptic ends of axons are a distance from nuclei, and maturing oocytes have large stores of maternal mRNAs. Certain stresses and mutations cause increased condensation of RNA binding proteins in *C. elegans* and *Drosophila* germ lines. In some cases, the condensates are liquid-like and reversible and have been hypothesized to be protective (Jud et al., 2008; Shimada et al., 2011; Hubstenberger et al., 2013); while in other cases, such as the *cgh-1 (tn691)* germ line at restrictive temperature, RNA binding proteins condense into sheet-like structures with immobile pools of protein (Hubstenberger et al., 2013; Langerak et al., 2019). The *cgh-1 (tn691)* phenotype also includes oogenesis defects, increased germ line apoptosis, and embryonic lethality; therefore, the pleiotropic nature of the defects makes it impossible to assess if the condensation of RNA binding proteins into solid structures contributes directly to the infertility (Navarro et al., 2001; Audhya et al., 2005). It will be of interest for future studies to find approaches to address the cause and effect relationships between condensate regulation and gamete development/function in the germ line.

## Author Contributions

JS contributed writing and editing of the manuscript and prepared the figures. ME contributed writing of the manuscript. Both authors contributed to the article and approved the submitted version.

## Conflict of Interest

The authors declare that the research was conducted in the absence of any commercial or financial relationships that could be construed as a potential conflict of interest.
